# Obesity among Medical Students of a Medical College: A Descriptive Cross-sectional Study

**DOI:** 10.31729/jnma.7519

**Published:** 2022-11-30

**Authors:** Vibina Aryal, Dayaram Ghimire, Sabita Kandel, Anirban Majumder, Sourav Manna

**Affiliations:** 1 Department of Physiology, National Medical College, Birgunj, Parsa, Nepal; 2 Department of Clinical Physiology, Maharajgunj Medical Campus, Maharajgunj, Kathmandu, Nepal

**Keywords:** *body fat*, *obesity*, *medical students*

## Abstract

**Introduction:**

The prevalence of overweight and obesity is increasing these days. The adverse effect of obesity can be seen in different physiological functions. Relative fat mass is a newly identified parameter to estimate whole body fat. This study aimed to find out the prevalence of obesity among medical students of a medical college.

**Methods:**

A descriptive cross-sectional study was carried out among medical students of a medical college from 1 September 2021 to 30 January 2022. Ethical approval was taken from the Institutional Review Committee (Reference number: FNMC/539/078/79). Simple random sampling was done. Height was measured using a stadiometer and waist circumference was measured using non-stretchable tape. Relative fat mass was calculated using the relative fat mass equation. The data was categorised according to the distribution of fat mass. Point estimate and 95% Confidence Interval were calculated.

**Results:**

Out of 180 medical students, 57 (31.67%) (24.87-38.47, 95% Confidence Interval) were obese according to relative fat mass cut-off. The mean fat mass among male and female participants with high relative fat mass was 27.057±1.42 and 35.674±2.63 respectively.

**Conclusions:**

The prevalence of obesity was lower than in other studies done in similar settings.

## INTRODUCTION

The prevalence of overweight and obesity has increased three times since 1975 worldwide.^1^ According to World Health Organization (WHO), among adults of age 18 and above, 39% were overweight and 13% were obese in 2016.^1^ A demographic and health survey done in Nepal in the year 2016 showed that 32.87% of women and 28.77% of men were overweight/obese.^2-4^

Relative fat mass (RFM) is a newly identified parameter to estimate whole-body fat.^5^ Studies have shown that RFM has a better diagnostic accuracy to define body fat when compared to Body Mass Index (BMI) which is a widely used parameter to define obesity in the clinical and public health sector.^6,7^

The objective of this study was to find out the prevalence of obesity among medical students of a medical college.

## METHODS

This was a descriptive cross-sectional study conducted among medical students of National Medical College (NMC), Birgunj, Nepal from 1 September 2021 to 30 January 2022. Ethical approval was taken from the Institutional Review Committee of the same institute (Reference number: FNMC/539/078/79). Students studying Bachelor of Medicine, and Bachelor of Surgery (MBBS) during the study period in NMC were included in the study. Informed consent was obtained from the study participants. The sample size was calculated using the following formula:


$$\begin{array}{ll} \text{n} & ={\text{Z}}^{2}\times \frac{\text{p}\times \text{q}}{{\text{e}}^{\text{2}}}\\ & =1.96^{2}\times \frac{0.50\times 0.50}{0.1^{2}}\\ & =97 \end{array}$$

Where,

n= minimum required sample size
Z= 1.96 at 95% Confidence Interval (CI)
p= prevalence taken as 50% for maximum sample size calculation
q= 1-p
e= margin of error, 10%

The sample was adjusted for finite population as follows:

n' = n / [1+{(n-1) / N}]

 = 97 / [1+{97-1) / 537}]

 = 83

Where,

n'= adjusted sample size
N= finite population of medical students in National Medical College, 537

The calculated minimum required sample size was 83.

A simple random sampling technique was used for data collection. A list of 537 medical students was made. The random number was assigned to all the students by using a random number function (RAND) in Microsoft Excel 2013. Then, a total of 180 students were selected from the list.

The height of the subjects was measured using a stadiometer and was recorded in centimetres. Waist circumference (WC) was measured with non-stretchable tape in a standing position at the end of expiration at the midpoint of the lower border of the ninth rib and the iliac crest.

RFM was calculated using the formula 64-(20 x heightWC) for males and 76-(20 x heightWC) for females.5 The calculated RFM was classified as essential fat (male: 2-5%, female: 10-13%), fitness (male: 14-17%, female: 21 to 24%), average (male: 18 to 24%, female: 25 to 31%) and obese (male: ≥ 25%, female: ≥ 32%).^8^

Data were then transferred into Excel 2013. Statistical Analysis was done using IBM SPSS Statistics 16.0. Point estimates and 95% CI were calculated.

## RESULTS

**Figure 1:**
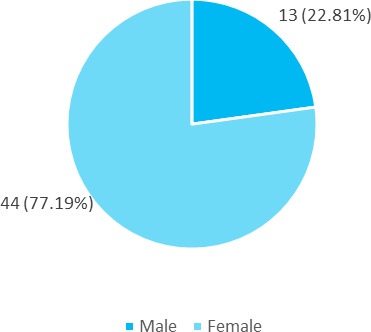
Distribution of RFM among male and female participants (n= 57).

Among 180 medical students, 57 (31.67%) (24.87-38.47, 95% CI) were obese according to relative fat mass cutoff. Among them 13 (22.81%) males and 44 (77.19%) females were obese according to RFM cut-off. (Figure 1).

The mean fat mass among male and female subjects with higher RFM was 27.057±1.42 and 35.674±2.63 respectively. Likewise, the mean waist circumference of male and female subjects with higher RFM was 91.538±5.190 and 76.841±5.70 respectively (Table 1).

**Table 1:** Baseline characteristics of total subjects with high RFM (n= 57).

Characteristics	Male (Mean ± SD)	Female (Mean ± SD)
RFM (%)	27.057±1.42	35.674±2.63
Height (cm)	168.908±8.35	154.295±5.60
Weight (Kg)	72.415±9.88	57.609±7.87
Waist circumference (cm)	91.538±5.190	76.841±5.70

## DISCUSSION

Our study showed that about 31.67 % medical students were obese according to RFM cut-off. This finding was slightly lower than the study done on the Korean population which found the prevalence of obesity as around 50%.^9^ The study done in a university-affiliated hospital in northern Israel among patients undergoing periodic examinations and in an urban slum of Karachi showed the prevalence of obesity to 83.70% and 89% respectively which is higher than our study.^6,10^ In our study, it was found that a greater proportion of obesity was seen in females than males which is similar to the other studies.^6,9,10^ RFM uses a simple model using two anthropometrics height and waist circumference.^5^ A study done on adult individuals who participated in National Health and Nutrition Examination Survey (NHNES) has validated this model by comparing body fat with dual-energy X-ray absorptiometry (DXA).^5^

The mean RFM in our study with high-fat mass was 27.057±1.42 in males and 35.674±2.63 in females. This result was consistent with another study in the Korean adult population where the mean RFM was 23.4±4.5 and 35.6±5.5 in male and female subjects respectively.^9^ Another study also had similar results where 27.9±5.^3^ and 40.5±5.9 was the mean RFM among male and female subjects respectively.^11^ Similarly, a study done on young male adults showed that the mean RFM among subjects with excess body fat was 25.2±3.7.^7^

Obesity is one of the emerging non-communicable diseases that has affected not just the adult population but also children and adolescents. Different studies have been conducted to find a better parameter to determine obesity that can represent the whole population.^5,12,13^ Different studies have shown that RFM correlates better with DXA and bioelectrical impedance (BIA) when compared with BMI.^7^ Another study showed that RFM had significantly better predictability of cardiometabolic risk compared to BMI.^6^

Our study was not able to show the effectiveness of RFM in predicting obesity-related health issues when compared to other anthropometric parameters like BMI that are widely used. As other parameters were not included in our study it is not possible to comment if this tool is better at predicting different risk factors. Large prospective studies are recommended to reveal the long-term clinical significance of RFM.

## CONCLUSIONS

The prevalence of higher RFM was lower than in other studies done in similar settings. However, body fat mass distribution was on the higher side so the appropriate preventive measures are recommended to prevent the negative consequences of high body fat mass.

## References

[1] (2021). World Health Organization. Obesity and overweight [Internet]..

[2] Ministry of Health, Nepal, New Era, ICF. (2017). Nepal demographic and health survey 2016 [Internet]..

[3] Rawal LB, Kanda K, Mahumud RA, Joshi D, Mehata S, Shrestha N (2018). Prevalence of underweight, overweight and obesity and their associated risk factors in Nepalese adults: Data from a Nationwide Survey, 2016.. PLoS One.

[4] Rana K, Ghimire P, Chimoriya R, Chimoriya R (2021). Trends in the prevalence of overweight and obesity and associated socioeconomic and household environmental factors among women in Nepal: Findings from the Nepal demographic and health surveys.. Obesities..

[5] Woolcott OO, Bergman RN (2018). Relative fat mass (RFM) as a new estimator of whole-body fat percentage — A cross-sectional study in American adult individuals.. Sci Rep..

[6] Kobo O, Leiba R, Avizohar O, Karban A (2019). Relative fat mass is a better predictor of dyslipidemia and metabolic syndrome than body mass index.. Cardiovasc Endocrinol Metab..

[7] Correa CR, Formolo NPS, Dezanetti T, Speretta GFF, Nunes EA (2021). Relative fat mass is a better tool to diagnose high adiposity when compared to body mass index in young male adults: A cross-section study.. Clin Nutr ESPEN..

[8] (2022). Relative Fat Mass (RFM) Calculator [Internet]..

[9] Paek JK, Kim J, Kim K, Lee SY (2019). Usefulness of relative fat mass in estimating body adiposity in Korean adult population.. Endocr J..

[10] Amin F, Fatima SS, Islam N, Gilani AH (2015). Prevalence of obesity and overweight, its clinical markers and associated factors in a high risk South-Asian population.. BMC Obes.

[11] Woolcott OO, Bergman RN (2020). Defining cutoffs to diagnose obesity using the relative fat mass (RFM): Association with mortality in NHANES 1999-2014.. Int J Obes (Lond)..

[12] Bergman RN, Stefanovski D, Buchanan TA, Sumner AE, Reynolds JC, Sebring NG (2011). A better index of body adiposity.. Obesity (Silver Spring)..

[13] Maessen MF, Eijsvogels TM, Verheggen RJ, Hopman MT, Verbeek AL, de Vegt F (2014). Entering a new era of body indices: the feasibility of a body shape index and body roundness index to identify cardiovascular health status.. PLoS One.

